# Therapeutic Potential of Rho Kinase Inhibitors in Corneal Disease: A Systematic Review of Preclinical and Clinical Studies

**DOI:** 10.3390/biomedicines13071602

**Published:** 2025-06-30

**Authors:** Laura Andreea Ghenciu, Diana Andrei, Claudia Borza, Roxana Iacob, Emil Robert Stoicescu, Sorin Lucian Bolintineanu, Daniela Iacob, Ovidiu Alin Haţegan

**Affiliations:** 1 Discipline of Pathophysiology, Department of Functional Sciences, “Victor Babes” University of Medicine and Pharmacy Timisoara, Square Eftimie Murgu 2, 300041 Timisoara, Romania; bolintineanu.laura@umft.ro; 2 Center for Translational Research and Systems Medicine, “Victor Babes” University of Medicine and Pharmacy Timisoara, Square Eftimie Murgu 2, 300041 Timisoara, Romania; 3 Department of Balneology, Medical Rehabilitation and Rheumatology, “Victor Babes” University of Medicine and Pharmacy Timisoara, Square Eftimie Murgu 2, 300041 Timisoara, Romania; 4 Centre of Cognitive Research in Pathological Neuro-Psychiatry NEUROPSY-COG, “Victor Babes” University of Medicine and Pharmacy Timisoara, Square Eftimie Murgu 2, 300041 Timisoara, Romania; 5 Department of Anatomy and Embriology, “Victor Babes” University of Medicine and Pharmacy Timisoara, Square Eftimie Murgu 2, 300041 Timisoara, Romania; roxana.iacob@umft.ro (R.I.); s.bolintineanu@umft.ro (S.L.B.); 6 Field of Applied Engineering Sciences, Specialization Statistical Methods and Techniques in Health and Clinical Research, Faculty of Mechanics, “Politehnica” University Timisoara, Mihai Viteazul Boulevard No. 1, 300222 Timisoara, Romania; stoicescu.emil@umft.ro; 7 Radiology and Medical Imaging University Clinic, “Victor Babes” University of Medicine and Pharmacy Timisoara, Square Eftimie Murgu 2, 300041 Timisoara, Romania; 8 Research Center for Pharmaco-Toxicological Evaluations, “Victor Babes” University of Medicine and Pharmacy Timisoara, Square Eftimie Murgu 2, 300041 Timisoara, Romania; 9 Department of Neonatology, “Victor Babes” University of Medicine and Pharmacy Timisoara, Square Eftimie Murgu 2, 300041 Timisoara, Romania; iacob.daniela@umft.ro; 10 Discipline of Anatomy and Embriology, Medicine Faculty, “Vasile Goldis” Western University of Arad, Revolution Boulevard 94, 310025 Arad, Romania; hategan.ovidiu@uvvg.ro

**Keywords:** Rho kinase inhibitors, Fuchs dystrophy, limbal stem cells deficiency, bullous keratopathy, ripasudil, netarsudil, ROCK inhibitors

## Abstract

**Background/Objectives:** Rho-associated coiled-coil-containing protein kinase inhibitors (ROCKis) have now become known as modulators of corneal endothelial wound repair and cell survival. However, evidence remains fragmented across laboratory and clinical reports. We performed a systematic review to synthesize preclinical and clinical data on ROCKis in corneal disease, assess their efficacy and safety, and identify research gaps. **Methods:** We searched PubMed, Web of Science, Scopus, and Google Scholar (until May 2025) for English-language original studies evaluating ROCKis in corneal models or patients. Inclusion criteria encompassed in vitro, ex vivo, in vivo, and clinical trials reporting functional outcomes (endothelial cell density, wound closure, visual acuity). **Results:** Thirty-one studies met criteria: 14 preclinical studies and 17 clinical studies. Preclinical models (rabbit, porcine, human explants) uniformly showed ROCKis (Y-27632, Ripasudil, Netarsudil, H-1152) accelerate corneal endothelial cell proliferation, migration, and restoration of a hexagonal monolayer with improved barrier and pump function over days to weeks. In 17 clinical investigations, topical Ripasudil or Netarsudil and cultured cell injections achieved significant corneal thinning, endothelial cell density and central corneal thickness changes, and visual acuity improvements (≥2 lines) with minimal adverse events. Overall bias was moderate in non-randomized studies and low in the RCTs. **Conclusions:** ROCKis demonstrate consistent pro-regenerative effects on corneal endothelium in multiple models and show promising clinical efficacy in Fuchs endothelial dystrophy and pseudophakic endothelial failure. Future work should explore novel delivery systems and larger controlled trials to optimize dosing, safety, and long-term outcomes.

## 1. Introduction

Rho-associated coiled-coil-containing protein kinases (ROCKs) are serine/threonine kinases that serve as key downstream effectors of the small guanosine triphosphatase (GTPase) Ras homolog family member A (RhoA) [1], regulating a variety of cellular processes such as cytoskeletal contractility, cell adhesion, motility, and membrane integrity [2]. By phosphorylating substrates such as myosin light-chain phosphatase (MLCP) and LIM kinase, ROCKs promote actomyosin-driven contraction and stress-fiber assembly, thereby regulating cell shape and tension across many tissue types [1,3]. Dysregulation of RhoA/ROCK signaling has been linked to a variety of diseases, including hypertension and fibrotic disorders, as well as delayed wound healing, making ROCKs a promising therapeutic target [4,5,6].

Small-molecule ROCK inhibitors, initially typified by the research compound Y-27632, have since evolved into clinically approved agents. Ripasudil (K-115), the first ROCK inhibitor approved for glaucoma in Japan, and Netarsudil (AR-13324), approved in the US for lowering intraocular pressure, both act by relaxing trabecular meshwork contractility to enhance aqueous outflow [7,8]. Beyond ocular hypertension, ROCK inhibition is now considered an effective modulator of endothelial cell behavior. It enhances the migration, proliferation, and barrier function of corneal endothelial cells (CECs) both in vitro and in vivo [9,10,11,12]. Additionally, preliminary clinical research indicates that it may hasten corneal clearance following Descemet’s stripping only (DSO) in Fuchs endothelial corneal dystrophy [13]. Current management of corneal endothelial diseases predominantly relies on surgical interventions. These procedures have significantly improved visual outcomes but remain technically demanding, require high-quality donor tissue, and carry risks such as graft failure, immunologic rejection, or detachment. In many regions, donor cornea shortages further limit access. Moreover, early or mild cases of endothelial dysfunction lack approved pharmacologic treatments, presenting a critical gap in non-surgical management [14,15].

Human corneal endothelial cells (CECs) are largely post-mitotic and rely on cell spreading and migration to maintain deturgescence [16]; in Fuchs endothelial corneal dystrophy (FECD), progressive endothelial loss leads to corneal edema and vision impairment [17]. Clinical and ex vivo studies demonstrate that, by attenuating RhoA-mediated contractility and activating Rac1-driven lamellipodial dynamics, topical ROCK inhibitors accelerate centripetal CEC migration across bare Descemet’s membrane, either after Descemet’s stripping only (DSO) or in wound-healing models, thereby shortening corneal clearance times, improving endothelial cell counts, and reducing the need for donor grafts [18,19]. Ongoing research focuses on improving delivery modalities, dosing regimens, and combination techniques to use ROCK inhibition as a pharmaceutical option or addition to transplantation in corneal endothelial dysfunction [20,21,22].

Despite these promising findings, the number of both laboratory and clinical investigations remains small and scattered. In this systematic review, we therefore aim to comprehensively gather and synthesize the existing preclinical and clinical evidence on ROCK inhibitors in corneal disease, assess their mechanisms of action, therapeutic efficacy, and safety profiles, and identify knowledge gaps to guide future research.

## 2. Materials and Methods

### 2.1. Search Strategy

We conducted a systematic literature search in PubMed, Google Scholar, Web of Science, and Scopus from the last 10 years (last database search 15 May 2025). Search terms combined variants of “Rho kinase inhibitor”, ”ROCK inhibitor”, “Y-27632”, “ripasudil”, “netarsudil”, “thiazovivin”, “H-1152”, “AR-13503”, “Y-39983”, “belumosudil“, with “cornea”, “corneal”, “endothelium”, “Fuchs dystrophy”, “cornea wound healing”, “limbal stem cell deficiency”, “bullous keratopathy”, and “pseudophakic endothelial failure”. Both MeSH headings and free-text keywords were used. Reference lists of all included studies and relevant reviews were hand-searched to capture any additional publications. Database search and query can be seen in the Supplementary Files (Table S2).

### 2.2. Eligibility Criteria

We included original preclinical (in vitro, ex vivo, in vivo) and clinical studies that evaluated at least one ROCK inhibitor in a corneal disease or injury model, reported functional/molecular/cellular outcomes (cell migration, proliferation, wound closure, endothelial cell density, clinical visual acuity, time to corneal clearance, safety/adverse events), and were published in English. We also included, since the evidence and research studies on ROCK inhibitors are scarce, case-reports and case-series. We excluded reviews, studies of ROCK inhibitors in non-corneal tissues, studies that reported only safety/adverse effects, and articles published in languages other than English. All inclusion/exclusion criteria can be accessed in Table S3.

### 2.3. Study Selection

**Figure 1:**
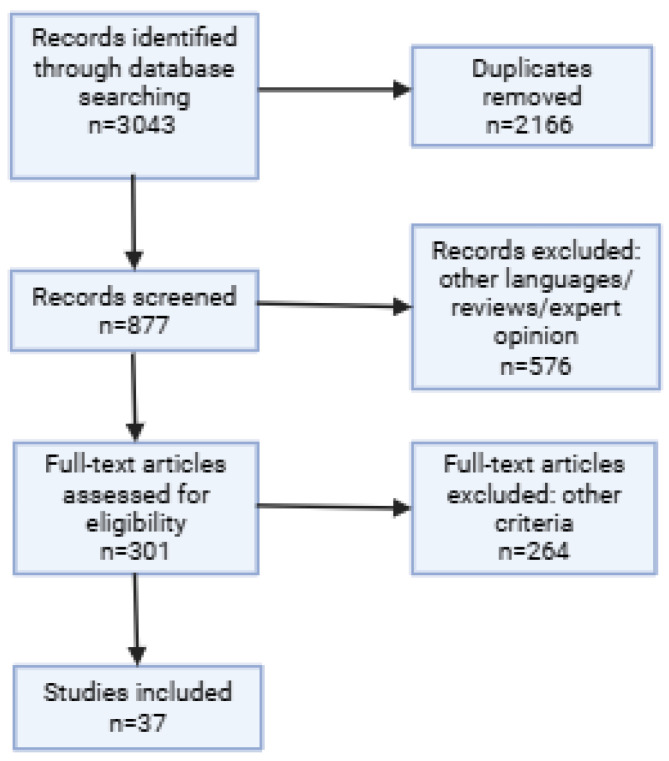
PRISMA flowchart.

Titles and abstracts were independently screened by two reviewers (C.B., S.L.B); disagreements were resolved by consensus with a third reviewer (L.A.G.). Full texts were then retrieved and assessed against the eligibility criteria. A PRISMA flow diagram was used to document the selection process (Figure 1).

### 2.4. Data Extraction and Synthesis

From each included study, two reviewers (D.A., L.A.G.) independently extracted the following: author, year, country, study design, model system (cell line, tissue, animal species, patient population), ROCK inhibitor tested and if it was used in conjunction with other local treatment/surgical intervention, primary and secondary outcomes, main findings, and any reported side-effects.

Given heterogeneity in models, inhibitors, and endpoints, we performed a narrative synthesis structured by study type (in vitro/ex vivo, animal, clinical) and by key outcomes/findings. We tabulated study characteristics and findings (mean/median, IQR, effect measures, where available) in evidence tables to facilitate comparison.

Randomized trials were assessed using the Cochrane Risk of Bias 2 (RoB 2) tool and non-randomized studies with the Risk of Bias in Non-randomized Studies—of Interventions (ROBINS-I) tool. Two reviewers (D.A., O.A.H.) independently rated each bias domain; any disagreements were resolved by discussion.

**Figure 2:**
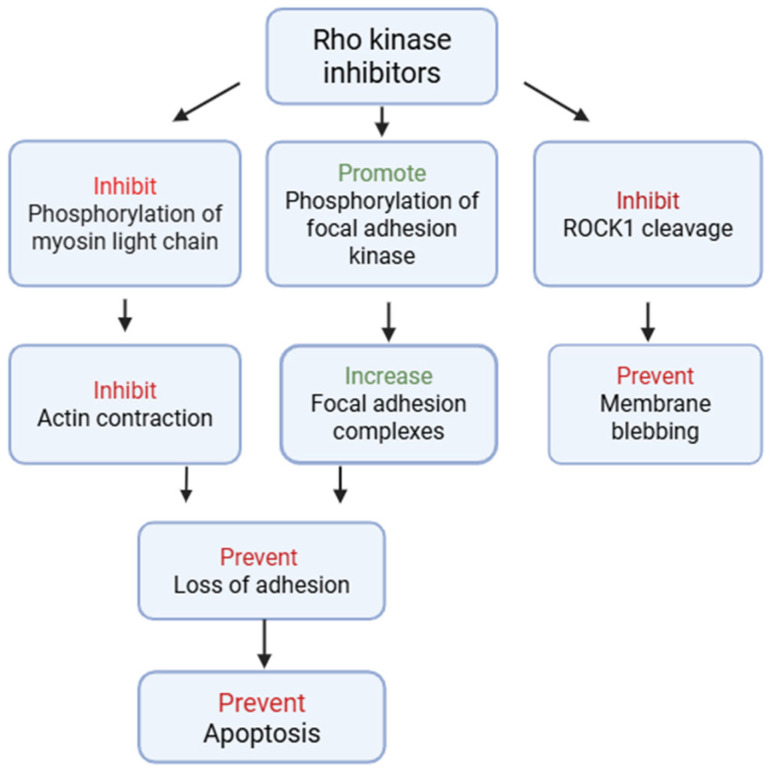
Mechanisms of action of Rho kinase inhibitors.

ROCK inhibitors exert their therapeutic benefits in corneal disease via a complex interaction of molecular and cellular mechanisms that go beyond their traditional involvement in cytoskeletal regulation (Figure 2). Their main mechanism of action is to modulate actomyosin contraction, which results in reduced stress fiber production and increased cellular plasticity. This property is vital in encouraging the adherence and propagation of corneal endothelial cells, particularly in regenerative techniques that require the re-establishment of a functioning monolayer [23]. ROCK inhibitors also prevent endothelial cell death by decreasing bleb formation, cellular contraction, and nuclear disintegration. (Figure 2) ROCK inhibition promotes cell cycle re-entry in normally quiescent CECs through cytoskeletal stress reduction [24]. This has been linked to enhanced proliferation through overexpression of cyclin D1 and downregulation of cell cycle inhibitors such as p27^Kip1 [25]. These effects promote the expansion of endothelial progenitor populations, both in vitro and in vivo, and contribute to cell-based therapies towards restoring corneal clarity. ROCK inhibitors have been demonstrated to reduce fibrotic remodeling in the corneal stroma by inhibiting TGF-β-mediated myofibroblast transformation. This anti-fibrotic impact reduces scarring and haze following injury or surgery while retaining the cornea’s biomechanical and optical qualities [5].

**Figure 3:**
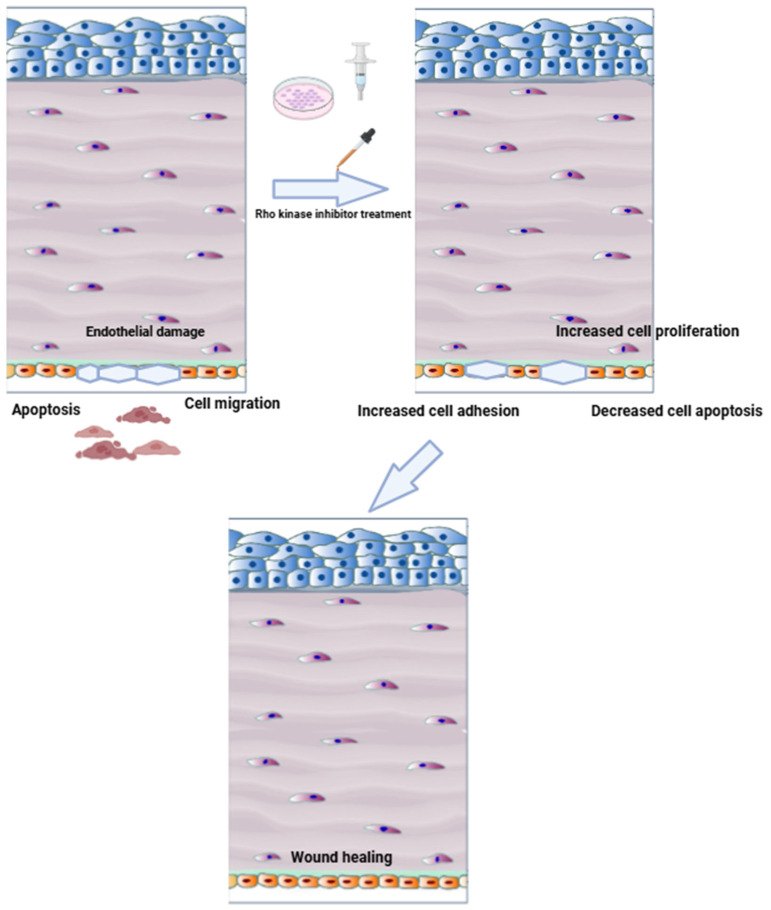
Rho kinase inhibitor treatment of corneal endothelial damage.

Emerging research also underlines ROCK inhibition’s antioxidative capability, which is especially important in Fuchs endothelial corneal dystrophy (FECD), where oxidative stress is a major cause of endothelial cell loss. ROCK inhibitors tend to stabilize mitochondrial function while decreasing reactive oxygen species (ROS) generation, hence improving cell survival under chronic stress [19]. Furthermore, they have shown anti-inflammatory properties. ROCK inhibitors diminish leukocyte adherence and cytokine expression in post-keratoplasty inflammation and endotheliitis models, most likely by inhibiting NF-κB signaling [26,27]. This helps to maintain a more stable immune system and facilitates tissue healing with fewer complications (Figure 3). Finally, ROCK inhibition protects the integrity of the endothelial barrier. They assist in retaining the corneal endothelium’s pump-and-barrier function, which is critical for preserving corneal clarity, by promoting tight junction protein production and inhibiting endothelial–mesenchymal transition (EndMT) [28].

## 3. Results

A total of 31 studies, which included 17 clinical investigations and 14 preclinical studies, have evaluated ROCK-inhibitor–based therapies. Clinical studies have been conducted across North America, Europe, Asia, and the Middle East, enrolling between 1 and 43 patients each (cumulatively over 200 subjects). The vast majority (7/16) originated in Japan, with additional cohorts in the United States (4 studies), Germany (1 study), Italy (1 study), Mexico (1 study), Saudi Arabia (1 study), Thailand (1 study), and Israel (1 study). Pathologies included Fuchs endothelial corneal dystrophy (7 studies), pseudophakic endothelial failure or bullous keratopathy (3 and 2 studies), and mixed or other endothelial disorders (5 studies). Eight preclinical investigations have been conducted across the UK, USA, Belgium, China, Germany, Japan, Singapore, and South Korea, using in vitro, ex vivo, and in vivo models.

### 3.1. Clinical Trials of ROCK Inhibitors in Corneal Endothelial Disease

**Figure 4:**
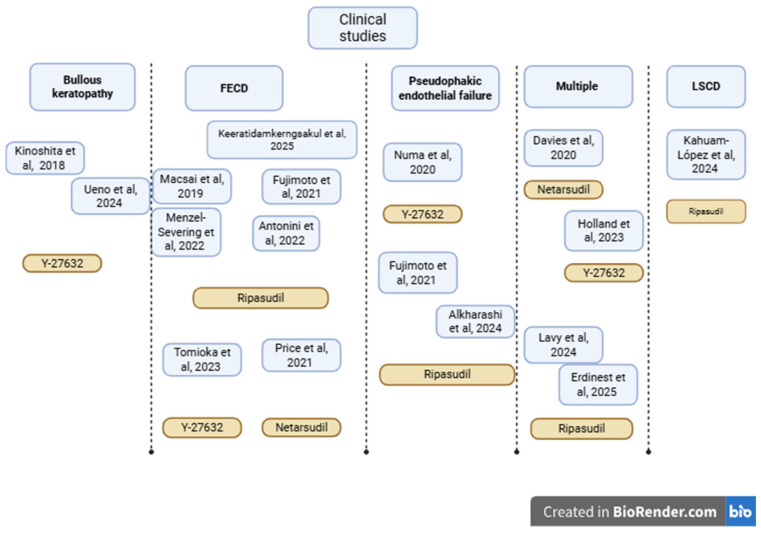
Concept map of clinical studies included in the review [13,29,30,31,32,33,34,35,36,37,38,39,40,41,42,43,44]. Created in BioRender.com. Ghenciu L.A. (2025).

Table 1 and Figure 4 summarize sixteen clinical investigations of Rho kinase–based therapies for corneal endothelial disorders, from in-human cell-injection studies to randomized drug trials and small case series.

#### 3.1.1. Study Designs, Interventions, and Disease Targets

Among the investigations, only one was a randomized double-masked trial, conducted by Price et al., which compared Netarsudil against placebo in 29 eyes with Fuchs endothelial dystrophy [32]. Four non-randomized prospective cohort studies evaluated Ripasudil in Fuchs dystrophy or pseudophakic patients [13,41] and administered cultured human corneal endothelial cells supplemented with Y-27632 [29,33]. Another prospective, open-label, single-arm clinical trial explored anterior chamber injection of cultured human CECs and Rho kinase inhibitor [29]. We also included three retrospective observational cohort studies which evaluated Ripasudil after pseudophakic failure and in Fuchs dystrophy [33,34,40]. The remaining six reports comprised small case series or single-patient case reports describing uses of Ripasudil or Netarsudil.

FECD was the most common indication (6 studies), followed by pseudophakic endothelial failure (3 studies), mixed endothelial injuries (4 studies), bullous keratopathy (2 studies), and limbal stem-cell deficiency (one study). Most trials required a low baseline endothelial cell density (<1000–1500 cells/mm^2^) or frank corneal edema.

Interventions comprised primarily topical administration of ROCK inhibitors, Ripasudil in ten studies and Netarsudil in two, and cell-based therapies consisting of surgical injection of cultured human corneal endothelial cells into the anterior chamber, co-administered with the ROCK inhibitor Y-27632.

#### 3.1.2. Outcomes and Efficacy

**Figure 5:**
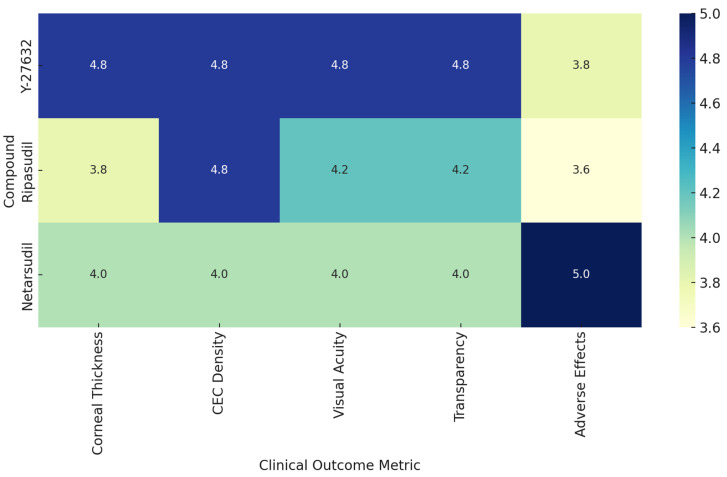
Key functional outcomes reported across included clinical studies.

In the two prospective cell-injection trials, Kinoshita et al. [29] achieved full corneal clarity by 24 weeks with mean central endothelial densities near 1900 cells/mm^2^ (10 of 11 eyes >1000 cells/mm^2^, six >2000 cells/mm^2^), 91 percent of eyes thinned below 630 µm, and 82 percent gained two or more lines of visual acuity, while Ueno et al. [41] reported a 94 percent success rate in reaching ≥1000 cells/mm^2^, an 82 percent edema-resolution rate, an average thickness reduction of 187 µm, and universal two-line best visual corrected acuity (BCVA) improvements. In the Ripasudil-augmented Descemet-stripping cohort, all treated eyes recovered 20/40 vision by two months and corneal thickness fell from roughly 700 µm preoperatively to the mid-500 µm range at one year, with central ECD rising from 860 to 1086 cells/mm^2^ by month 12. Among topical ROCK-inhibitor studies in Fuchs dystrophy or pseudophakic endothelial failure, Fujimoto’s retrospective series demonstrated a stable three percent reduction in both CCT and TCT at one month and negligible ECD change [33,34], Alkharashi’s comparative cohort showed a 4.5 percent ECL versus 12.8 percent in controls at 12 months with preserved vision [42], while Price et al. found significant corneal thinning (–20 µm at one month, –26 µm at three months) and a 1.6-line scotopic VA gain versus placebo [32]. Ripasudil also significantly preserved central ECD and minimized paracentral ECD loss (0.4% vs. 7.3%), with stable corneal densitometry and no significant difference in CCT changes compared to controls [44]. Small case series and reports of Ripasudil and Netarsudil described resolution of edema and visual improvement in 1–4 patients. Figure 5 presents a heatmap comparing the clinical efficacy of three major Rho-associated kinase inhibitors (Y-27632, Ripasudil, and Netarsudil) across five core clinical outcome metrics: corneal thickness, CEC density, visual acuity, corneal transparency, and adverse effects. Each compound was scored from 0 to 5, with higher values indicating more favorable outcomes based on aggregated findings from relevant studies. Table 2 exhibits primary and secondary outcomes and results of the studies.

### 3.2. Preclinical Investigations of ROCK Inhibitors

#### 3.2.1. Experimental Models and Approaches

**Figure 6:**
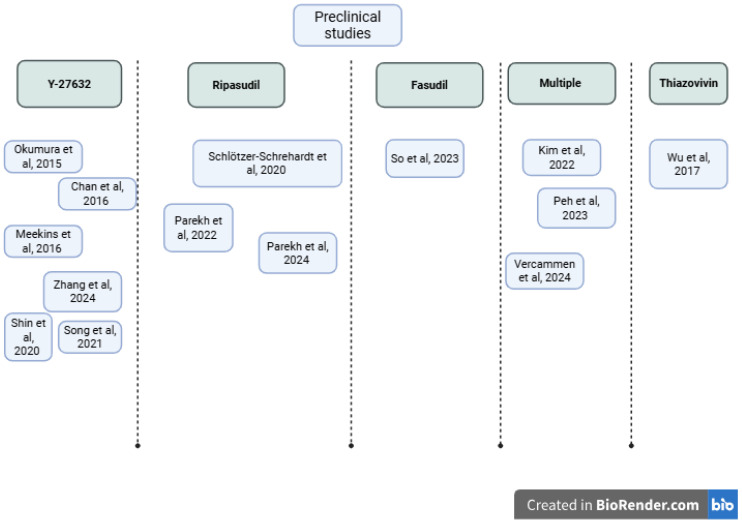
Concept map of preclinical studies included in the review [9,18,45,46,47,48,49,50,51,52,53,54,55,56]. Created in BioRender.com, Ghenciu L.A. (2025).

We identified 14 preclinical investigations of Rho kinase inhibitors using in vivo, ex vivo, and in vitro models (Table 3, Figure 6). In vivo rabbit models showed that topical Y-27632 dramatically increases endothelial cell cycling and restores corneal clarity. After mechanical scrape injury, Ki67+ nuclei rose from approximately 13% to 65% with 10 mM Y-27632 drops, actin corticalization was normalized, and stromal edema virtually disappeared by two weeks [45]. Similarly, in the rabbit scrape model, H-1152 eye drops accelerated wound closure, led to faster corneal thinning, and also showed histologic evidence of re-endothelialization with fewer stress fibers and more hexagonal cells compared to saline controls [9].

Ex vivo human explant study shows cultured FECD Descemet–endothelial complexes treated with Ripasudil show broad transcriptional shifts, downregulation of stress-fiber genes and upregulation of cell-cycle drivers, adhesion receptors, and barrier/pump proteins, coupled with sustained PCNA/Ki67 elevation for at least 72 h [46]. In organ-culture wounds, Ripasudil boosts Ki67+ cells from ~12% to ~48% at the wound edge whether Descemet’s membrane remains or is stripped and leads to a hexagonal monolayer on stroma in both cases.

Quantitative ex vivo migration assays also highlight Ripasudil’s capability; the analysis on human FECD and normal Descemet’s membranes revealed a roughly two- to three-fold increase in cell velocity and displacement within hours of 1 µM Ripasudil [47]. In another study, Ripasudil doubled both migration speed and distance at the wound border and early stroma, and when combined with fibronectin–chondroitin sulfate (FNC)-coating, achieved up to 3× velocity, while promoting late-stage zonula occludens-1 (ZO-1) re-monolayering and a sustained Ki67 surge at 60 days [18].

In vitro investigations demonstrated that ROCK inhibition is beneficial for corneal endothelial progenitor isolation [48] and that Ripasudil preserves viability up to 10 µM while showing 5-bromo-2′-deoxyuridine (BrdU)/Ki67 proliferation in immortalized FECD lines. A broader screening of six chemically distinct ROCK-i classes (Y-27632, Netarsudil, AR-13503, verosudil, Ripasudil, and Y-39983) by real-time impedance identified AR-13324 and AR-13503 as comparably or more stimulatory than Y-27632 at low nanomolar doses, with differential cytotoxicity profiles at higher concentrations [49]. Treatment with Thiazovivin at 4 µM was determined to be optimal in the study of Wu et al., leading to improved cell morphology and tight junctions, increased expression of ZO-1 and Na⁺/K⁺-ATPase, decreased ROCK1/2 expression, and decreased Snail expression [50].

In the study of Vercammen et al. [51], the interventions included agents such as Ripasudil, Thiazovivin, Fasudil, Y-27632, Chroman-1, and SAR407899. The outcomes demonstrated that several of these ROCK inhibitors (Ripasudil, Y-27632, SAR407899, Thiazovivin, Fasudil, ROCKi-2, and Chroman-1) enhanced cell proliferation and wound closure over five days without exhibiting cytotoxicity. However, Thiazovivin and SR-3677 were associated with aberrant tight junction morphology as indicated by fragmented ZO-1 expression. Most inhibitors preserved the expression of ZO-1 and Na⁺/K⁺-ATPase, essential for endothelial barrier and pump function, although Y-27632 slightly reduced ZO-1 levels. Furthermore, key cell cycle regulators including p16, CDK2, and Cyclin E1 were not significantly altered, suggesting that the proliferative effects did not result in uncontrolled or dysregulated cell division. Another study demonstrated that both sovesudil and PHP-0961, novel ROCK inhibitors, significantly improved cell proliferation, adhesion, and wound healing in hCEnCs, with effects equal to or greater than the benchmark ROCK inhibitor Y-27632 [52].

Ripasudil disassembles stress fibers, downregulates endothelial–mesenchymal transition (EnMT) drivers, and upregulates Rac1 activity approximately 1.7–2.1× over baseline to nucleate actin-related protein 2/3 (Arp2/3)-dependent lamellipodia, correlating tightly with increased leading-edge ARPC2/actin colocalization and accelerated wound closure [18].

Additionally, mucoadhesive hydroxypropyl methylcellulose–polyethylene glycol (HPMC-PEG)-carbopol films loaded with Y-27632 not only remain adherent under washout but deliver 2–3× more drug into porcine corneas (including cryoprobed lesions) over 6 h than equivalent eye-drop solutions [37]. Real-time impedance and proliferation assays compared six ROCK inhibitors (Y-27632, Ripasudil, Netarsudil, AR-13503, verosudil, Y-39983). AR-13324 and AR-13503 were effective at low nanomolar doses, though with variable cytotoxicity at higher concentrations [49]. Ripasudil and Y-27632 were among the most studied and consistently effective compounds.

**Table 3:** Summary of preclinical investigations of Rho kinase inhibitors.

Study, Year	Country	Sample Size and Model	Study Design	Experimental Approach	Intervention
Okumura et al., 2015 [45]	Japan	9 rabbits; 3 humans	In vivo/clinical	Rabbit wound model; human case series	10 mM Y-27632 drops
Chan et al., 2016 [53]	UK	12 porcine corneas	In vitro	Mucoadhesive film deposition	HPMC-PEG 400 films with Y-27632
Meekins et al., 2016 [9]	USA	6 human post-mortem eyes; 6 rabbits	In vivo/ex vivo	IHC; scratch/migration; in vivo scrape + H-1152 drops	Y-27632 (in vitro/ex vivo); H-1152 (in vivo)
Wu et al., 2017 [50]	China	6 human donor corneas	In vitro	Culture of primary and passaged HCECs	Thiazovivin at various concentrations (2, 4, 6 µM); comparison with Y-27632 as positive control
Schlötzer-Schrehardt et al., 2020 [46]	Germany	FECD explants (*n* = 450); wounds (*n* = 30); intact (*n* = 20); cell lines (*n* = 3)	In vitro/ex vivo	Organ-culture wound model	Ripasudil
Shin et al., 2020 [48]	South Korea	10 human corneal tissues	In vitro	HCEP culture and differentiation	10 µM Y-27632
Song et al., 2021 [54]	China	PCECs (from 4 pigs)/7 rabbits	In vitro/in vivo	PCEC primary culture; ICC (NSE); MTT assay; EdU assay; rabbit CED model;	Y-27632 at 0–200 µM
Kim et al., 2022 [52]	South Korea	hCEnCs/porcine corneas	In vitro/ex vivo	hCEnC culture; BrdU, Ki67, scratch assay, ICC, WB, Mito assays	Y-27632 (control), sovesudil, PHP-0961
Parekh et al., 2022 [47]	USA	Biological triplicates.	Ex vivo	Live-cell migration on DM	Ripasudil 1 µM
Peh et al., 2023 [49]	Singapore	32 cadaver corneas	In vitro/ex vivo	EDM explant + primary CEC assays	Y-27632, Netarsudil, AR-13503, verosudil, ripasudil, Y-39983
So et al., 2023 [55]	South Korea	20 rabbits	In vivo/in vitro	iPSC; NCC; CEC differentiation; rabbit CED model	Fasudil 10 µM
Parekh et al., 2024 [18]	USA	Biological triplicates	Ex vivo	TrackMate; scratch; Rac1 assays	Ripasudil 1 µM
Vercammen et al., 2024 [51]	Belgium	Biological triplicates	In vitro	MTS, scratch assay, proliferation (OrBITS), ICC, WB	ROCKi library (15 compounds including Y-27632, Ripasudil, Thiazovivin, Fasudil; 50 μM–5 nM)
Zhang et al., 2024 [56]	China	Rats/B4G12 human CEC line	In vivo/in vitro	hCEC culture; TGF-β1-induced EndMT; EDU assay; scratch assay; ICC; WB; qPCR	Y-27632 0.2 μM

Abbreviations: CEC—corneal endothelial cell; DM—Descemet’s membrane; EDM—endothelium–Descemet’s membrane complex; FECD—Fuchs endothelial corneal dystrophy; HCEP—human corneal endothelial progenitor; HPMC—Hydroxypropyl Methylcellulose; IHC—immunohistochemistry; PCECs—porcine corneal endothelial cells; PEG—Polyethylene Glycol; Rac1—Ras-related C3 Botulinum toxin substrate 1.

#### 3.2.2. Summary of Preclinical Findings

Across these preclinical models, ROCK inhibition consistently accelerated endothelial recovery, it drove a several-fold rise in proliferative markers, doubled to tripled migration speed and distance, and restored a hexagonal, pump- and barrier-competent monolayer.

### 3.3. Risk of Bias

Across the clinical studies, the dominant source of bias was inadequate control for confounding, such as a lack of comparator arms or adjustment for baseline differences. Participant selection was also a concern due to potential referral biases. Outcome measurement and selective reporting were largely low risk, though a few studies showed moderate selective reporting. Overall, most non-randomized trials carry a moderate risk of bias under ROBINS-I. The three randomized trials exhibited low risk across randomization, adherence, outcome measurement, and reporting, resulting in an overall low risk of bias under RoB 2 (Table 4 and Table 5). For the animal studies, we used the SYRCLE Risk of Bias tool to assess methodological quality across ten domains (D1–D10). Many items were rated as unclear, especially those related to randomization and blinding procedures. These concerns led to an overall risk of bias rated as moderate to high for most studies (Table 6).

We did not include case reports and series in this risk assessment as any conclusions drawn from this single-patient report must be viewed with caution, since without a control group or randomization it is impossible to know whether the observed improvements reflect the treatment itself, the natural course of disease, or other factors. Reporting on just one person also risks drawing inferences from an unrepresentative case, and assessments made by clinicians aware of the intervention can introduce observer bias.

## 4. Perspectives

Although Rho kinase inhibitors have already demonstrated a definite advantage in facilitating the migration of corneal endothelial cells and wound repair, their therapeutic potential goes well beyond Fuchs dystrophy and several important research gaps remain unaddressed. These gaps limit the broader clinical translation and long-term impact of ROCK-based therapies in ophthalmology. Most studies to date have targeted well-established indications such as Fuchs dystrophy or bullous keratopathy, with limited efforts to evaluate ROCK inhibitors in earlier stages of corneal endothelial dysfunction, post-keratoplasty complications, or other corneal disorders with fibrotic or inflammatory components. An important gap is the limited exploration of combinatory therapeutic approaches. ROCK inhibitors may have synergistic potential when combined with other agents, such as anti-inflammatory or anti-fibrotic drugs. Furthermore, combining ROCK inhibition with cell-based therapies has shown promise in improving cell adhesion, proliferation, and survival. However, rigorous clinical trials evaluating such strategies are lacking, and existing studies continue to focus on monotherapies rather than multimodal interventions. From a mechanistic perspective, the downstream effects of ROCK inhibition in human corneal tissue are not yet fully elucidated. Although their role in cytoskeletal modulation and cell junction integrity is well described, little is known about how ROCKs interact with other key pathways involved in corneal healing.

ROCK inhibition also shows promise for posterior segment disorders. Netarsudil’s increased lipophilicity and tissue penetration profile have prompted its evaluation for intravitreal delivery. AR-13503, a Netarsudil prodrug, has been engineered as a biodegradable, sustained-release intravitreal implant in preclinical studies [57] and is now in clinical development for diabetic macular edema and neovascular age-related macular degeneration (NCT03835884). Additionally, intracapsular delivery approaches have been explored; intraocular lenses coated with poly(lactic-*co*-glycolic acid) (PLGA) loaded with the ROCK inhibitor Y-27632 have demonstrated efficacy in inhibiting lens epithelial cell proliferation and preventing posterior capsular opacification in preclinical models [58].

Currently, topical ocular solutions Ripasudil and Netarsudil are FDA-approved ROCK inhibitors [59]. The most common and practical method of delivering ROCK inhibitors is through topical treatments; however, permeability and absorption difficulties restrict their effectiveness. In the future, outpatient treatment of chronic endothelial dysfunction may be made possible by sustained-release formulations such as mucoadhesive films, nanoparticle carriers, or in situ gelling eye drops, which could sustain therapeutic concentrations on the ocular surface for longer.

**Figure 7:**
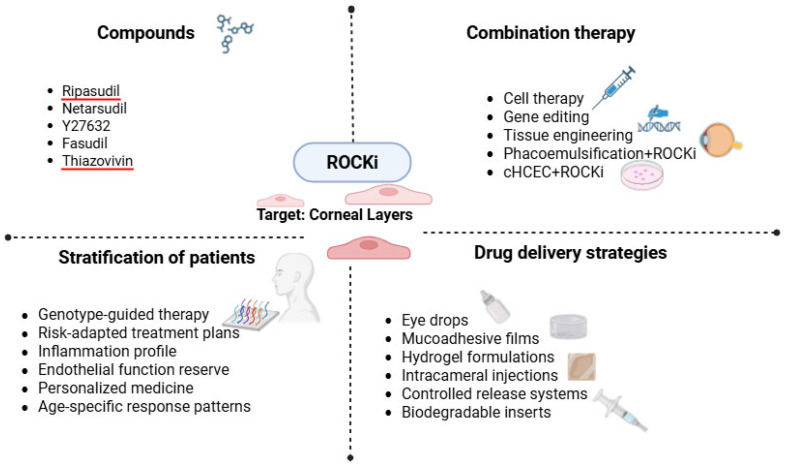
Preclinical to clinical utility of ROCK inhibitors in corneal diseases.

Chitosan-based nanoparticles (Cs NPs) were developed as a mucoadhesive carrier for the hydrophilic ROCK inhibitor Fasudil, addressing its inherently low ocular bioavailability. Their findings indicate that chitosan nanoparticles can significantly improve Fasudil’s ocular retention, permeation, and safety profile [60]. Additionally, intravitreal depot approach could deliver continuous ROCK inhibition to outflow pathways and benefit glaucoma patients [61]. Combining ROCK inhibitors with cell-based therapies further boosts engraftment and survival of transplanted cells, while pairing ROCK blockade with anti-inflammatory or anti-fibrotic agents could address the multifactorial pathogenesis of severe corneal diseases. Figure 7 shows current and possible applications of Rho kinase inhibitors in corneal diseases.

We also conducted a search for clinical trials for both completed and ongoing interventional studies of ROCK inhibitors in human corneal endothelial diseases. In a Phase 1 trial, 22 adults with corneal edema from endothelial dysfunction received intrastromal injections of HCEC-1 combined with low, mid or high doses of the ROCK inhibitor Y-27632 to evaluate safety and preliminary effects on corneal thickness and vision (NCT05309135). In another prospective cohort study at University Hospital Dubrava, 50 adults with both Fuchs endothelial corneal dystrophy and glaucoma undergoing cataract surgery have been assigned to receive either topical Netarsudil 0.02% or a placebo once daily for 30 days postoperatively. The primary endpoint is change in ECD measured by non-contact specular microscopy at 37 days, with visual acuity as a secondary outcome (NCT06969586). In the same hospital, 50 adults with both glaucoma and pseudophakic bullous keratopathy will be randomized to receive either a once-daily fixed-dose combination of Netarsudil 0.02%/Latanoprost 0.005% or a placebo for three months following lens implantation. Visual acuity and central corneal thickness will be assessed at baseline and bi-weekly thereafter to determine whether topical ROCK inhibition with concomitant prostaglandin analog alleviates corneal edema and improves vision compared to placebo (NCT06960629).

In DETECT I and II, 160 adults with endothelial dysfunction (primary Fuchs dystrophy, pseudophakic edema, and prior graft failure) have been randomized in a 2×2 factorial design across seven U.S. centers to undergo either ultrathin DSAEK or DMEK and to receive topical Ripasudil 0.4% or placebo, with masked assessment of visual acuity and endothelial cell loss over 12 months (NCT05289661). In another single-center, triple-masked Phase IIa trial at the University of Erlangen-Nürnberg, 21 adults with moderate to advanced Fuchs endothelial corneal dystrophy undergo Descemetorhexis and are randomized to receive either topical Ripasudil 0.4% or preservative-free artificial tears for three months. The primary endpoint is the incidence of serious adverse reactions within three months; secondary outcomes include all adverse events, changes in endothelial cell density and corneal thickness, BCVA, contrast sensitivity, and the need for rescue DMEK (NCT03575130).

The last registered trial included a Phase II, double-blind trial at the Devers Eye Institute, where 72 adults with Fuchs endothelial corneal dystrophy undergoing DMEK are randomized to receive either Ripasudil 0.4% eye drops or preservative-free artificial tears six times daily for 2–4 weeks postoperatively. The primary endpoint is time to corneal clearance over six months, with secondary assessments of BCVA, endothelial cell density, and rates of graft detachment or failure (NCT03813056).

Despite the fact that some of these trials enroll significantly larger cohorts than previous pilot studies, they are still limited to well-established routes, such as topical drops or intrastromal/cellular injections, and do not explore truly novel delivery methods or completely new therapeutic modalities for corneal endothelial disease.

## 5. Limitations

Several limitations temper the strength of our conclusions. Most clinical studies enrolled small cohorts, often in single centers, limiting generalizability. There was also a substantial heterogeneity in interventions, dosages, and outcome measures, which precluded quantitative meta-analysis. Follow-up durations were often short, leaving long-term safety and durability of effect uncertain. Finally, preclinical models also varied widely and the translational relevance of some endpoints to clinical outcomes remains to be fully validated.

## 6. Conclusions

Rho kinase inhibition accelerates corneal endothelial repair through multiple complementary pathways. In preclinical models, ROCK inhibitors induce cell-cycle re-entry, dismantle stress fibers, and activate Rac1/Arp2/3-driven lamellipodia to boost migration, and restore a hexagonal, barrier- and pump-competent monolayer. Topical Ripasudil or Netarsudil have been shown to increase endothelial cell density, reduce central corneal thickness, and improve best-corrected visual acuity by ≥2 Snellen lines with minimal side effects. Additional multicenter, randomized studies are required to optimize dose regimens, assess long-term safety, and develop standardized efficacy outcomes.

## Figures and Tables

**Table 1:** Summary of clinical studies evaluating ROCK inhibitor therapies.

Study, Year	Country	Design	Patients	Indication	Intervention
Kinoshita et al., 2018 [29]	Japan	Uncontrolled, single group study	11 patients	Bullous keratopathy	Surgical transfer of cHCECs with a Rho-associated kinase inhibitor (Y-27632) into the anterior chamber
Macsai et al., 2019 [13]	USA	Prospective, non–placebo-controlled clinical trial	18 patients	FECD	Ripasudil after Descemet stripping only
Numa et al., 2020 [30]	Japan	Prospective observational study	11 patients	Pseudophakic endothelial failure	cHCECs supplemented with Rho-associated protein kinase inhibitor (Y-27632)
Davies et al., 2020 [31]	USA	Case series	4 patients	Multiple	Netarsudil
Price et al., 2021 [32]	USA	Prospective, double masked RCT	29 patients	FECD	Netarsudil
Fujimoto et al., 2021 [33]	Japan	Retrospective observational study	26 patients (33 eyes)	Pseudophakic endothelial failure	Ripasudil
Fujimoto et al., 2021 [34]	Japan	Retrospective observational study	24 patients	FECD	Ripasudil
Menzel-Severing et al., 2022 [35]	Germany	Case series	3 patients	FECD	Transcorneal freezing and Ripasudil
Antonini et al., 2022 [36]	Italy	Case series	3 patients	FECD	Ripasudil
Holland et al., 2023 [37]	USA	Prospective, randomized, double-masked study	22 patients	Multiple	Intracameral injection of CECs + Y-27632
Tomioka et al., 2023 [38]	Japan	Case report	1 patient	FECD	Transcorneal freezing and topical Rho kinase inhibitor
Kahuam-López et al., 2024 [39]	Mexico	Case report	1 patient	LSCD	Ripasudil
Lavy et al., 2024 [40]	Japan	Single-center retrospective cohort study	16 patients	Multiple	Ripasudil
Ueno et al., 2024 [41]	Japan	Multicenter, double-blind physician-initiated phase II clinical trial	29 eyes	Bullous keratopathy	Surgical transfer of cHCECs with a Rho-associated kinase inhibitor (Y-27632) into the anterior chamber
Alkharashi et al., 2024 [42]	Saudi Arabia	Prospective, non-randomized, non-blinded comparative study	43 patients	Pseudophakic endothelial failure	Ripasudil
Erdinest et al., 2025 [43]	Israel	Case series	3 patients	Multiple	Ripasudil
Keeratidamkerngsakul et al., 2025 [44]	Thailand	Randomized controlled trial	31 patients	FECD	Ripasudil

Abbreviations: cHCEC—cultured human corneal endothelial cell; FECD—Fuchs endothelial corneal dystrophy; LSCD—limbal stem cell deficiency; RCT—randomized clinical trial.

**Table 2:** Key efficacy and safety outcomes from clinical studies of ROCK-based interventions.

Indication	Study	Primary Outcomes	Secondary Outcomes	Key Efficacy Results	Additional Outcomes	Safety
**Bullous keratopathy**	[29]	Corneal transparency, CEC density >500 cells/mm^2^	Corneal thickness <630 µm, BCVA +2 lines	Corneal transparency 100%; CEC mean 1924 cells/mm^2^ (range 947–2833)	CT <630 μm in 91% (mean 549 μm); BCVA ↑ ≥2 lines in 82%	No adverse effects
	[41]	CEC density ≥1000 cells/mm^2^ at 24 weeks	Corneal thickness, edema, BCVA	94.1% achieved target CEC; BCVA 100%; CCT improved; edema resolved	CT <630 μm in 82.4%; ΔCT −187.4 μm; BCVA ↑ in 100%	Eye pain (33.3%), IOP ↑ (14.8%)
**Fuchs distrophy**	[32]	Change in central corneal thickness	CDVA, FECD disability score	CCT reduced by 26 µm at 3 months; CDVA +1.6 lines	Scotopic CDVA ↑ by +1.6 lines; no change in disability score	One withdrawal due to epithelial bullae
	[13]	Pachymetry, ECD, VA to 20/40	ECD >1000, BCVA ≥20/50, haze-free	All achieved 20/40 VA by 2 months; CCT and ECD improved	-	No adverse effects
	[34]	LogMAR VA, CCT, TCT		Mean VA: 0.024; CCT: 0.972; TCT: 0.970 at 1 month	-	Not assessed
	[37]	Safety	Change in CCT and BCVA	CCT ↓ 133.4 µm (–17.74%); BCVA ↑ 0.662 logMAR; 89% had ≥3-line improvement	BAT ↑ 0.97 logMAR; ECD ↑ ~823 cells/mm^2^; no dose–response	Mild/moderate TEAEs; transient IOP ↑ (5/22); 1 unrelated SAE
	[44]	Central ECD loss at 3 months	Paracentral ECD loss, CCT change ratio	ECD ↑ by 145 cells/mm^2^; paracentral loss ↓ to 0.4%; CCT stable	Central ECD ↑ by +145 cells/mm^2^; paracentral loss ↓ to 0.4%; stable densitometry	Transient conjunctival erythema
**Pseudophakic endothelial failure**	[42]	CCT and ECD	BCVA	CCT change: 0.9%; ECD loss: 4.5%; BCVA improved from 0.70 to 0.15 logMAR	BCVA ↓ from 0.70 → 0.15 LogMAR at 12 months	No adverse effects
	[30]	ECD, CCT, BCVA	IOP, CV, % hexagonality	ECD 91%; BCVA improved to 0.046 logMAR; CV and % hexagonality improved	No IOP change; CV ↓ from 0.46 → 0.37; Hexagonality ↑ from 47% → 54%	Not assessed
	[33]	TCT, CCT, and ECD	-	TCT and CCT ratios improved; ECD loss reduced from 14.1% to –4.5%	-	Not assessed

Abbreviations: BAT—brightness acuity testing; BCVA—best corrected visual acuity; CCT—central corneal thickness; CDVA—corrected distance visual acuity; ECD—endothelial cell density; IOP—intraocular pressure; TCT—thinnest corneal thickness.

**Table 4:** Risk-of-bias assessment for non-randomized clinical studies using the ROBINS-I tool.

Study	Confounding	Selection of Participants	Classification of Intervention	Deviations from Intended Interventions	Missing Data	Measurement of Outcomes	Selection of Reported Results	Overall
Ueno et al. [41]	Serious	Moderate	Low	Low	Moderate	Low	Low	Moderate
Macsai et al. [13]	Serious	Moderate	Low	Moderate	Low/moderate	Low	Low	Moderate
Alkharashi et al. [42]	Serious	Moderate	Low	Moderate	Low	Low	Low	Moderate
Fujimoto et al. [33]	Low/moderate	Low	Low	Low	Moderate	Low	Low	Low
Lavy et al. [40]	Low/moderate	Low	Low	Low	Moderate	Low	Low	Low
Fujimoto et al. [34]	Moderate/serious	Serious	Low	Moderate	Moderate	Low	Low	Moderate

**Table 5:** Risk-of-bias assessment for the randomized clinical trial using the Cochrane RoB 2 tool.

Study	Randomization Process	Deviations from Intended Interventions	Missing Outcome Data	Measurement of the Outcome	Reported Results	Overall
Price et al. [32]	Low	Low	Moderate	Low	Low	Low
Holland et al. [37]	Low	Low	Low	Low	Low	Low
Keeratidamkerngsaku et al. [44]	Low	Low	Low	Low	Low	Low

**Table 6:** Risk-of-bias assessment for animal studies using SYRCLE.

Study	D1	D2	D3	D4	D5	D6	D7	D8	D9	D10	Overall
Okumura et al. [45]	Unclear	Low	Unclear	Unclear	Low	Unclear	Unclear	Low	Low	Low	Low/Moderate
Meekins et al. [9]	Unclear	Low	Unclear	Unclear	Low	Unclear	Unclear	Low	Low	Low	Moderate
Song et al. [54]	Unclear	Low	Unclear	Unclear	Unclear	Unclear	Unclear	Low	Low	High	Moderate/High
So et al. [55]	Unclear	Low	Unclear	Unclear	Unclear	Unclear	Unclear	Low	Unclear	High	Moderate/High
Zhang et al. [56]	Unclear	Low	Unclear	Unclear	Unclear	Unclear	Unclear	Low	Low	Low	Moderate

Legend: D1—sequence generation; D2—baseline characteristics; D3—allocation concealment; D4—random housing; D5—blinding of caregivers/investigators; D6—random outcome assessment; D7—blinding of outcome assessor; D8—incomplete outcome data; D9—selective outcome reporting; D10—other sources of bias.

## Data Availability

Data available from the corresponding authors.

## Supplementary Materials

The following supporting information can be downloaded at https://www.mdpi.com/article/10.3390/biomedicines13071602/s1, Table S1: PRISMA Checklist (Source: ref. [62]). Table S2. Search strategy and query results for ROCK inhibitors in corneal disease research. Table S3. Inclusion and exclusion criteria.
