# Supplementary material for: Therapeutic Potential of Rho Kinase Inhibitors in Corneal Disease: A Systematic Review of Preclinical and Clinical Studies
Source: Biomedicines. 2025 Jun 30;13(7):1602. doi: 10.3390/biomedicines13071602 (PMC12292609; doi:10.3390/biomedicines13071602)
Supplement: Supplementary file 1 [file biomedicines-13-01602-s001.zip › Tables S2 and S3.pdf]

Table S2. Search Strategy and Query Results for ROCK Inhibitors in Corneal Disease Research

**Query**	**Results**
((((((((cornea) OR (corneal)) OR (endohelium)) OR (fuchs distrophy)) OR (limbal stem cell deficiency)) OR (pseudophakic failure)) OR (bullous keratopathy)) OR (cornea wound healing)) AND (rho kinase inhibitor)	1 150
((((((((cornea) OR (corneal)) OR (endohelium)) OR (fuchs distrophy)) OR (limbal stem cell deficiency)) OR (pseudophakic failure)) OR (bullous keratopathy)) OR (cornea wound healing)) AND (ripasudil)	350
((((((((cornea) OR (corneal)) OR (endohelium)) OR (fuchs distrophy)) OR (limbal stem cell deficiency)) OR (pseudophakic failure)) OR (bullous keratopathy)) OR (cornea wound healing)) AND (rock inhibitor)	945
((((((((cornea) OR (corneal)) OR (endohelium)) OR (fuchs distrophy)) OR (limbal stem cell deficiency)) OR (pseudophakic failure)) OR (bullous keratopathy)) OR (cornea wound healing)) AND (thiazovivin)	9
((((((((cornea) OR (corneal)) OR (endohelium)) OR (fuchs distrophy)) OR (limbal stem cell deficiency)) OR (pseudophakic failure)) OR (bullous keratopathy)) OR (cornea wound healing)) AND (Y-27632)	349
((((((((cornea) OR (corneal)) OR (endohelium)) OR (fuchs distrophy)) OR (limbal stem cell deficiency)) OR (pseudophakic failure)) OR (bullous keratopathy)) OR (cornea wound healing)) AND (netarsudil)	186
((((((((cornea) OR (corneal)) OR (endohelium)) OR (fuchs distrophy)) OR (limbal stem cell deficiency)) OR (pseudophakic failure)) OR (bullous keratopathy)) OR (cornea wound healing)) AND (fasudil) 42/25 ((((((((cornea) OR (corneal)) OR (endohelium)) OR (fuchs distrophy)) OR (limbal stem cell deficiency)) OR (pseudophakic failure)) OR (bullous keratopathy)) OR (cornea wound healing)) AND (H-1152)	18
((((((((cornea) OR (corneal)) OR (endohelium)) OR (fuchs distrophy)) OR (limbal stem cell deficiency)) OR (pseudophakic failure)) OR (bullous keratopathy)) OR (cornea wound healing)) AND (AR-13503)	5
((((((((cornea) OR (corneal)) OR (endohelium)) OR (fuchs distrophy)) OR (limbal stem cell deficiency)) OR (pseudophakic failure)) OR (bullous keratopathy)) OR (cornea wound healing)) AND (Y-39983)	31

Table S3. Inclusion and exclusion criteria

**Criteria Type**	**Description**
Inclusion Criteria	- Original preclinical studies (in vitro, ex vivo, in vivo) or clinical studies<br>- Evaluated at least one ROCK inhibitor in a corneal disease or injury model<br>- Reported at least one functional outcome (cell migration, proliferation, wound closure, endothelial cell density) or clinical outcomes (visual acuity, corneal clearance time) or molecular/cellular outcomes (expression of tight junction/pump proteins, apoptosis, fibrosis markers)<br>- Published in English<br>- Case reports and case series were included due to limited available evidence<br>- Studies published between 2016-2025 (last ten years)
Exclusion Criteria	- Review articles<br>- Studies investigating ROCK inhibitors in non-corneal tissues<br>- Articles published in languages other than English<br>- Studies that evaluated only safety/adverse effects of ROCK inhibitors<br>- Studies that evaluated ROCK inhibitors for other ocular/non-ocular diseases
